# 3种液相色谱-串联质谱法检测脂溶性维生素的性能确认和一致性评价

**DOI:** 10.3724/SP.J.1123.2025.04002

**Published:** 2026-03-08

**Authors:** Bingchu LI, Binghui LI, Zhiyang CHEN, Ziyang LI, Meiling YIN, Xing LYU, Zhongyuan XIANG, Qichen LONG, Min HU

**Affiliations:** 1.中南大学湘雅二医院检验医学科，湖南 长沙 410011; 1. Department of Laboratory Medicine，the Second Xiangya Hospital of Central South University，Changsha 410011，China; 2.分子诊断技术湖南省工程研究中心，湖南省感染性疾病分子诊断临床医学研究中心，湖南省临床分子诊断中心，湖南 长沙 410011; 2. Molecular Diagnostic Technology Hunan Engineering Research Center，Clinical Medical Research Center for Molecular Diagnosis of infectious diseases in Hunan Province，Center for Clinical Molecular Diagnostics in Hunan Province the Second Xiangya Hospital of Central South University，Changsha 410011，China

**Keywords:** 脂溶性维生素, 液相色谱-串联质谱, 一致性, 校准, fat-soluble vitamins, liquid chromatography-tandem mass spectrometry （LC-MS/MS）, consistency, calibration

## Abstract

精准测定脂溶性维生素A、D、E、K（VA、25（OH）D、VE、VK）水平对健康评估与疾病诊断具有重要价值，液相色谱-串联质谱法（LC-MS/MS）因其高特异性与灵敏度成为其主要检测手段，但方法间结果的可比性与一致性仍待提升。本研究依据CLSI C62及《液相色谱-质谱临床应用建议》，对3种LC-MS/MS方法检测脂溶性维生素的线性范围、检出限、定量限、精密度和准确度等进行系统评估。研究选取中南大学湘雅二医院于2022年8月至10月期间留存的40例剩余血清样本，采用3种LC-MS/MS方法进行脂溶性维生素检测。通过Passing-Bablok回归分析评估3种方法之间的相关性，绘制Bland-Altman图分析其相对偏差，并以一致性相关系数（CCC）评价3种方法检测结果的一致性。同时，比较不同方法在重新校准前后的检测一致性。研究结果显示，3种LC-MS/MS方法的总变异系数（CV）为2.7%~10.1%，加标回收率为88.8%~109.2%，表明其检测性能符合临床应用要求。一致性分析结果显示，其中两种方法在25（OH）D检测中表现出中等一致性（CCC=0.938），其余LC-MS/MS方法的一致性相关系数范围为0.322~0.853，存在一定差异。经统一校准品重新校准后，除VK外，其余脂溶性维生素的检测一致性明显改善，CCC提高至0.918~0.983。综上所述，3种LC-MS/MS方法在检测脂溶性维生素方面具备良好的性能，但方法间一致性尚存不足。通过使用统一校准品进行重新校准，可显著提升检测结果的一致性。本研究为分析脂溶性维生素临床质谱检测方法的一致化现状及其优化路径提供了数据支持，有助于提高检测结果的准确性与可靠性，从而为临床诊断与治疗提供更坚实的依据。

## Abstract (en)

Accurate quantification of fat-soluble vitamin A （VA）， vitamin D （25（OH）D）， vitamin E （VE）， vitamin K （VK） is essential for health assessment and disease diagnosis. Liquid chromatography-tandem mass spectrometry （LC-MS/MS） plays a crucial role in the measurement of these vitamins due to its high specificity and sensitivity. However， the comparability and concordance of the detection results among different LC-MS/MS methods are still suboptimal. In this study， the analytical performance including linearity， limit of detection， limit of quantitation， precision， accuracy and carryover was evaluated according to CLSI C62 and *the Suggestions on Clinical Application of Liquid Chromatography-Mass Spectrometry*. Aliquots of 40 patient samples were collected from August to October 2022 from the Second Xiangya Hospital， Central South University. These fat-soluble vitamins were measured using three LC-MS/MS methods. Passing-Bablok regression， Bland-Altman plots and concordance correlation coefficient （CCC） were performed to compare the differences among three methods. The consistency of the results before and after recalibration with a unified calibrator was evaluated. The results showed that the total CVs for the three mass spectrometry kits were 2.7%-10.1%， and the recoveries for all kits ranged from 88.8% to 109.2%， indicating that their detection performance met the requirements for clinical application. Concordance correlation analysis showed that two of the three methods had substantial agreement in vitamin D testing （CCC： 0.938）， while the CCCs for other methods ranged from 0.322 to 0.853. After recalibration with a unified calibrator， except for vitamin K， the CCCs for the results of the three methods ranged from 0.918 to 0.983. In conclusion， the performance validation results of three LC-MS/MS methods for fat-soluble vitamins meet clinical requirements. The consistency among the three methods was poor， but using a unified calibrator significantly improved the consistency of these methods. The study provides recommendations for the standardization of clinical fat-soluble vitamin testing， thereby contributing to improved accuracy and consistency of results. This study will provide more reliable evidence for clinical diagnosis and treatment.

脂溶性维生素包括维生素A、D、E和K（VA、25（OH）D、VE、VK），在人体生理功能中发挥至关重要的作用，广泛参与免疫调节、视力维持、骨骼代谢等生理过程^［1］^。其中血清中维生素D的主要循环代谢形式为25-羟基维生素D₂（25（OH）D₂）和25-羟基维生素D₃（25（OH）D_3_）。总25（OH）D浓度是指这两者之和，被广泛认为是评估个体25（OH）D营养状态的关键生化指标^［2，3］^。此外，这些维生素还被视为多种疾病的潜在监测指标，其异常水平与孤独症、佝偻病、妊娠期糖尿病及哮喘等疾病密切相关^［4，5］^。因此，脂溶性维生素的精准检测对个体健康状况的评估和诊断具有重要的临床价值。目前临床实验室中常用免疫法检测25（OH）D^［6］^，高效液相色谱法（HPLC）-紫外/可见光或光电二极管阵列检测器测定VA和VE^［7，8］^，色谱法检测VK^［9］^，但这些方法存在无法多指标联合检测、样品前处理步骤复杂、提取时间长的问题，且方法灵敏度与准确性不高。相比之下，液相色谱-串联质谱（LC-MS/MS）技术在高效液相色谱法的基础上结合具有高精密度和高灵敏度特点的质谱检测仪，能够快速、准确地检测样本中脂溶性维生素^［10］^。

近年来脂溶性维生素的质谱测定技术发展迅速，但不同实验室及方法之间在操作流程、校准体系和参考标准方面的差异，仍然是影响检测结果可比性的主要因素。Zhou等^［11］^比较了不同临床实验室使用LC-MS/MS检测血清25（OH）D的结果，结果表明，通过优化LC-MS/MS方法显著提高了血清25（OH）D的检测一致性；Qin等^［12］^比较了3种方法在血浆儿茶酚胺和甲氧基肾上腺素测量中的一致性，结果显示，肾上腺素和去甲肾上腺素的结果在不同试剂盒方法之间的一致性较差，而变肾上腺素和去甲变肾上腺素的结果一致性较好。不同研究表明不同方法使用的校准品、色谱柱、样品前处理条件及适配的质谱仪存在较大差异，缺乏规范化的操作流程。当前，临床实验室普遍使用不同质谱检测平台，但跨平台间缺乏系统化的方法学一致性评估，限制了质谱检测结果在临床中的标准化应用。近年来，国际和国内已建立25（OH）D的参考检测方法^［13，14］^，显著提升了质谱法检测25（OH）D结果的准确性和一致性^［15，16］^，但VA、VE、VK等脂溶性维生素仍缺乏标准化参考体系，相关检测方法之间的差异性尚未得到系统评估。因此，评价检测不同脂溶性维生素的LC-MS/MS方法在临床应用中的性能，明确其可比性和一致性，对于推动脂溶性维生素检测的标准化和规范化具有重要意义。

本研究拟采用液相色谱-串联质谱检测平台，依据CLSI C62 ^［17］^和《液相色谱-质谱临床应用建议》^［18］^对3种LC-MS/MS方法（分别用于测定5种脂溶性维生素）进行性能验证，并评估3种LC-MS/MS方法检测脂溶性维生素结果的一致性。

## 1 实验部分

### 1.1 仪器与试剂

Acquity UPLC I-Class/Xevo TQ-S液相色谱-串联质谱仪、96孔正压装置（美国Waters公司）；96孔板氮吹仪（天津博纳艾杰尔科技有限公司）；涡旋振荡器（中国Kylin Bell公司）；超纯水仪（美国Millipore公司）；小型高速冷冻离心机、多功能台式离心机（德国Eppendorf公司）。

方法A：脂溶性维生素同位素内标（VA-d_6_、25（OH）D_2_-d_3_、25（OH）D_3_-d_3_、VE-d_6_和VK-d_4_），校准品（S1~S6），质控品（QC1、QC2）干粉，含正己烷的萃取液，含有异丙醇、乙腈、水、甲酸的复溶液和流动相均购自国内广州市丰华生物工程有限公司（批号：20222400603），校准品可溯源至NIST SRM 968f。

方法B：脂溶性维生素同位素内标品（VA-d_4_、25（OH）D_2_-d_3_、25（OH）D_3_-d_5_、VE-d_6_、VK-d_7_）、校准品、质控品溶液、稀释液、复溶液、萃取液购自湖南豪思生物科技有限公司（批号：20202400103），其中VA校准品可溯源至Cerilliant V-011，25（OH）D_2_、25（OH）D_3_校准品可溯源至NIST 2972b，VE校准品可溯源至Cerilliant V-020，VK校准品可溯源至Cerilliant V-030。

方法C：脂溶性维生素同位素内标品（VA-d_6_、25（OH）D_2_-d_6_、25（OH）D_3_-d_3_、VE-d_6_、VK-d_4_）、校准品、质控品（QC I、QC Ⅱ）干粉、含正己烷的提取液、含甲酸和甲酸铵的甲醇水溶液的复溶液、含牛血清白蛋白的校准品稀释液和流动相购自山东英盛生物技术有限公司（批号：20212401390），其中VA校准品可溯源至Cerilliant V-011，25（OH）D_2_、25（OH）D_3_校准品可溯源至NIST 2972a，VE校准品可溯源至Cerilliant V-020，VK校准品可溯源至Cerilliant V-030。

### 1.2 实验方法

#### 1.2.1 标准溶液的配制

方法A：在使用前将1 mL纯化水加入校准品瓶中（S1~S6）中，彻底混匀，直到完全溶解。方法B：用甲醇将高浓度校准品溶液按等比系列稀释，获得7个浓度梯度的混合标准工作溶液。方法C：以校准品稀释液对高浓度校准品溶液进行等比系列稀释，获得7个浓度梯度的混合标准工作溶液。不同方法配制浓度如表1所示。

**表1:** 不同方法中标准溶液的质量浓度 (ng/mL)

Method	VA	25（OH）D_2_	25（OH）D_3_	VE	VK
A	30	1.14	2.2	880	0.10
	60	2.28	4.4	1760	0.20
	135	5.70	11.0	4400	0.55
	300	11.40	22.0	8800	1.00
	600	22.80	44.0	17600	1.85
	1600	57.00	110.0	44000	5.00
B	10.25	1.04	1.05	96	0.10
	20.50	2.07	2.10	192	0.21
	48.50	5.18	5.25	480	0.49
	102.51	10.36	10.49	960	1.03
	205.03	20.72	20.98	1920	2.05
	1025.15	103.61	104.90	9600	10.25
	2050.29	207.22	209.80	19570	20.50
C	25	1.56	3.13	625	0.09
	50	3.13	6.25	1250	0.19
	100	6.25	12.50	2500	0.38
	200	12.50	25.00	5000	0.75
	400	25.00	50.00	10000	1.50
	800	50.00	100.00	20000	3.00
	1600	100.00	200.00	40000	6.00

VA： vitamin A； 25（OH）D： 25-hydroxy vitamin D； VE： vitamin E： VK： vitamin K.

#### 1.2.2 研究对象基本信息

本研究中3种方法性能验证部分所用的血清样本来自中南大学湘雅二医院检验科的剩余血清，通过将上述单人份样本混合为血清池后添加或不添加标准溶液制备而成；用于方法学比对的样本来自中南大学湘雅二医院健康管理中心2022年8月至10月的40例表观健康者的剩余血清样本，统计学和基线资料见表2。分装为3等份后-80 ℃冻存。每个样本均使用3种方法进行分析。本研究已获得中南大学湘雅二医院伦理委员会的批准，审批号为LYF2022229。

**表2:** 健康人群的人口学特征（*n*=40）

Characteristics	Healthy individuals
Age/year	34.5 （27.0， 42.0）
Gender/（female/male）	20/20
BMI/（kg/m^2^）	20.2±1.7
ALT/（U/L）	17.0 （10.2， 21.4）
AST/（U/L）	23.0±5.1
GLU/（mmol/L）	4.6±0.6
TG/（mmol/L）	0.9±0.3
Cre/（μmol/L）	71.0 （58.0， 84.8）
UA/（μmol/L）	268.7±69.6

Normally， distributed data are presented as mean±standard deviation. Nonnormally distributed data are presented as median （25th， 75th percentile）. BMI： body mass index； ALT： alanine aminotransferase； AST： aspartate aminotransferase； GLU： glucose； TG： triglyceride； Cre： creatinine； UA： uric acid.

排除标准：（1）年龄<18岁或>70岁；（2）近1个月内服用维生素滴剂、抗凝药、降脂药等可能影响体内维生素水平的药物；（3）血常规、肝肾功能、尿常规、粪便隐血结果异常者；（4）患有血液系统疾病、泌尿系统疾病、消化系统疾病、风湿免疫性疾病、循环系统疾病、内分泌和遗传代谢性疾病、恶性肿瘤、传染性疾病或正在接受输血治疗或肿瘤放化疗等；（5）溶血、脂血、黄疸标本；（6）处于妊娠期。

#### 1.2.3 样本处理

方法A：取200 μL血清样本于相应的2 mL离心管中，均加入200 μL脂溶性维生素同位素内标，涡旋混匀后加入1 000 μL萃取液，在2 000~2 400 r/min下旋涡振荡10 min，于-20 ℃下静置10 min，13 000 r/min离心5 min；取上层液体800 μL转移到96孔深孔板中，室温下氮气吹干；加入200 μL复溶液，在2 000~2 400 r/min下旋涡振荡5 min，进行复溶。将96孔深孔板放置于离心机中3 000~3 500 r/min离心5 min后进行检测。

方法B：取100 μL血清样本，分别转移至96孔U型板中，加入100 μL内标工作液，涡旋混匀1 min。加入600 μL萃取液，振荡混匀3~5 min后，于4 ℃、4 000 r/min离心15 min。收集400 μL上清液转移至另一块96孔U型板中，氮气吹干后加入100 μL复溶液，振荡混匀3~5 min。再次于4 ℃、4 000 r/min离心5 min，取90 μL上清液转移至350 μL 96孔V型板中进行检测。

方法C：取300 μL血清样本加入至96孔提取板中，加入200 μL内标工作液，吹打混匀。取400 μL混合液转移至配有96孔收集板的96孔过滤板中，使用正压装置将样品压入过滤孔内，静置5~10 min。加入750 μL含正己烷的提取液，采用正压方式收集洗脱液至收集板中，并重复该洗脱步骤一次。将收集板置于室温下，通过氮气吹干残留溶剂后，加入100 μL复溶液，振荡混匀2 min。最终将全部溶液转移至96孔进样板中进行检测。

#### 1.2.4 分析条件

方法A分析条件如下，流动相A：含甲酸的甲醇水溶液，流动相B：含甲酸的甲醇溶液；进样量：5 μL；流速：0.50 mL/min；色谱柱：C18色谱柱（50 mm×2.1 mm，2.6 μm）；柱温：40 ℃；梯度洗脱程序：0~0.50 min，70%B；0.50~3.00 min，70%B~100%B；3.00~4.20 min，100%B；4.20~4.30 min，100%B~70%B；4.30~5.00 min，70%B。

方法B分析条件如下，流动相A：含甲酸水溶液，流动相B：含甲酸、乙酸铵的甲醇溶液；进样量：5 μL；流速：0.25 mL/min；色谱柱：C18色谱柱（30 mm×3.0 mm，2.6 μm）；柱温：40 ℃；梯度洗脱程序：0~1.80 min，70%B~90%B；1.80~2.50 min，90%B~99%B；2.50~4.00 min，99%B；4.00~4.10 min，99%B~70%B；4.10~5.50 min，70%B。

方法C分析条件如下，流动相A：含甲酸、甲酸铵的水溶液，流动相B：含甲酸、甲酸铵的甲醇溶液；进样量：2 μL；流速：0.40 mL/min；色谱柱：C18色谱柱（50 mm×2.1 mm，3.0 μm）；柱温：40 ℃；梯度洗脱程序：0~1.00 min，60%B；1.00~4.00 min，60%B~98%B；4.00~7.00 min，98%B；7.00~8.50 min，98%B~60%B；8.50~9.00 min，60%B。

离子源：电喷雾离子（ESI）源，正电离模式；扫描模式：多反应监测（MRM）模式；离子源温度：150 ℃；脱溶剂气流量：1 000 L/h；碰撞气：氩气。毛细管电压：3.5 kV（方法A和B），3.0 kV（方法C）；脱溶剂气温度：500 ℃（方法A和B），550 ℃（方法C）。质谱参数详见表3。

**表3:** 3种LC-MS/MS方法的质谱参数

Method	Compound	Parent ion （*m/z*）	Daughter ion （*m/z*）	CV/V	CE/eV
A	VA	269.2	93.1	80	10
	25（OH）D_2_	395.3	119.1	42	28
	25（OH）D_3_	383.4	257.2	40	14
	VE	431.3	165.1	80	6
	VK	451.4	187.1	55	22
	VA-d_6_	275.2	96.1	80	24
	25（OH）D_2_-d_3_	398.3	121.1	42	21
	25（OH）D_3_-d_3_	386.3	214.1	40	25
	VE-d_6_	437.3	171.2	50	10
	VK-d_4_	455.4	191.1	55	22
B	VA	269.2	93.1	20	40
	25（OH）D_2_	395.3	107.1	20	22
	25（OH）D_3_	383.3	257.1	20	12
	VE	431.3	83.1	20	70
	VK	451.4	187.1	40	22
	VA-d_4_	273.2	94.1	20	20
	25（OH）D_2_-d_3_	398.3	380.3	20	8
	25（OH）D_3_-d_5_	406.3	388.4	20	8
	VE-d_6_	437.3	171.3	20	70
	VK-d_7_	458.4	194.1	40	22
C	VA	269.2	93.0	20	50
	25（OH）D_2_	413.4	113.1	20	20
	25（OH）D_3_	383.3	257.1	20	14
	VE	431.3	83.1	20	80
	VK	451.4	187.1	40	22
	VA-d_6_	275.2	96.0	20	20
	25（OH）D_2_-d_6_	419.2	337.2	20	6
	25（OH）D_3_-d_3_	404.3	368.1	20	10
	VE-d_6_	437.3	171.3	20	30
	VK-d_4_	455.4	191.1	40	22

CV： cone voltage； CE： collision energy.

## 2 结果与讨论

### 2.1 方法学验证

#### 2.1.1 线性范围、检出限和定量限

配制3种LC-MS/MS方法的系列混合标准工作溶液并进样分析，以目标物的质量浓度为横坐标（*x*，ng/mL），以目标物与对应内标的定量离子峰面积之比为纵坐标（*y*），绘制标准曲线。结果见表4。3种LC-MS/MS方法检测脂溶性维生素在线性范围内相关性良好，相关系数（*R*）为0.994 5~0.999 6；以3倍信噪比计算检出限（LOD），10倍信噪比计算定量限（LOQ）。方法A的LOD为0.03~88 ng/mL，LOQ为0.10~880 ng/mL；方法B的LOD为0.02~20 ng/mL，LOQ为0.05~60 ng/mL；方法C的LOD为0.01~39 ng/mL，LOQ为0.02~78 ng/mL，各方法能够满足脂溶性维生素的测定需求。不同方法的代表性色谱图见图1。

**表4:** 3种LC-MS/MS方法检测脂溶性维生素的线性范围、相关系数、检出限和定量限

Compound	Method	Linear range/（ng/mL）	*R*	LOD/（ng/mL）	LOQ/（ng/mL）
VA	A	30-1600	0.9948	3	30
	B	10.25-2050.29	0.9952	1.27	4.15
	C	25-1600	0.9995	3	12
25（OH）D_2_	A	1.14-57.00	0.9961	0.38	1.01
	B	1.04-207.22	0.9976	0.28	0.92
	C	1.56-100.00	0.9956	0.10	0.39
25（OH）D_3_	A	2.20-110.00	0.9963	0.73	2.20
	B	1.05-209.80	0.9977	0.23	0.73
	C	3.13-200.00	0.9982	0.20	0.39
VE	A	880-44000	0.9947	88	880
	B	96-19570	0.9945	20	60
	C	625-40000	0.9996	39	78
VK	A	0.10-5.00	0.9952	0.03	0.10
	B	0.10-20.50	0.9949	0.02	0.06
	C	0.09-6.00	0.9991	0.01	0.02

**图1:**
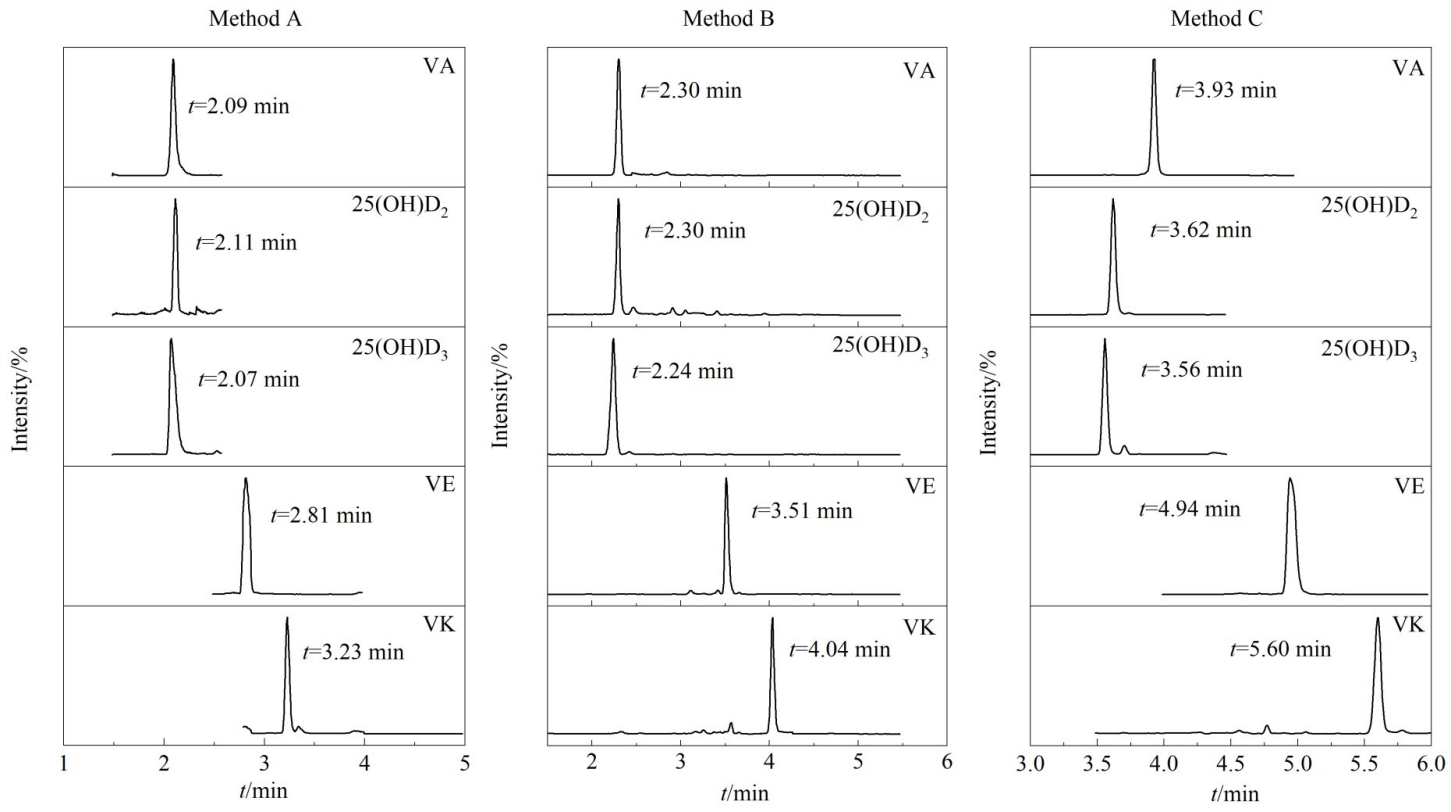
采用不同方法时的代表性色谱图

#### 2.1.2 精密度

为了考察不同LC-MS/MS方法的精密度，参考CLSI C62^［17］^和《液相色谱-质谱临床应用建议》^［18］^，在低、中、高3个不同水平下，分别使用制备好的混合血清做5个平行5天重复的精密度验证，计算变异系数（CV），批内CV=（批内标准差/批内平均值）×100%，批间CV=（批间标准差/批间平均值）×100%，总CV=（总标准差/总平均值）×100%。结果如表5所示，3种LC-MS/MS方法的批内CV为2.0%~4.1%（VA）、2.1%~7.4%（25（OH）D_2_）、2.9%~6.4%（25（OH）D_3_）、2.5%~5.2%（VE）和3.0%~6.2%（VK），批间CV为1.7%~4.7%（VA）、3.9%~7.0%（25（OH）D_2_）、3.7%~10.0%（25（OH）D_3_）、1.2%~6.4%（VE）、1.7%~8.7%（VK），总CV为2.7%~6.0%（VA）、6.7%~9.8%（25（OH）D_2_）、4.7%~10.4%（25（OH）D_3_）、3.8%~7.9%（VE）和4.6%~10.6%（VK），符合《液相色谱-质谱临床应用建议》^［18］^中对精密度的要求（CV<15%）。上述结果说明3种LC-MS/MS方法具有良好的精密度，均可满足临床检测的需求。

**表5:** 3种LC-MS/MS方法检测脂溶性维生素在3个水平下的精密度（*n*=5）

Compound	Method	Low level				Medium level				High level			
		*C*/（ng/mL）	Inter-run CV/%	Intra-run CV/%	Total CV/%	*C*/（ng/mL）	Inter-run CV/%	Intra-run CV/%	Total CV/%	*C*/（ng/mL）	Inter-run CV/%	Intra-run CV/%	Total CV/%
VA	A	306.2	4.1	4.4	6.0	626.2	2.2	3.6	4.2	826.5	2.0	1.5	2.5
	B	205.7	4.3	3.6	5.6	358.3	2.7	4.7	5.5	509.6	2.9	3.1	4.2
	C	225.8	2.0	1.7	2.7	399.4	2.5	2.0	3.2	571.3	2.0	1.9	2.8
25（OH）D_2_	A	3.2	7.0	6.9	9.8	5.6	5.5	5.2	7.6	9.2	2.2	6.9	7.2
	B	2.1	7.4	4.2	8.5	2.9	6.8	3.9	7.8	5.4	5.4	3.9	6.7
	C	2.2	4.7	7.0	8.4	3.1	5.9	5.4	8.0	5.9	4.2	6.2	7.5
25（OH）D_3_	A	10.5	3.4	6.7	7.5	19.8	4.6	4.6	6.5	45.6	3.3	4.7	5.7
	B	12.2	3.7	5.9	6.9	22.9	3.6	4.2	5.6	54.1	5.4	4.9	7.3
	C	11.4	2.9	3.7	4.7	22.1	6.4	4.2	7.6	50.8	3.0	10.0	10.4
VE	A	6311.7	4.1	3.9	5.6	11540.4	4.2	6.4	7.6	21581.9	4.3	2.0	4.8
	B	4577.3	3.9	4.1	5.6	9565.9	5.3	5.9	7.9	15567.6	4.4	5.5	7.1
	C	4960.9	2.5	2.9	3.8	9338.5	4.6	1.2	4.8	17704.0	4.3	2.8	5.1
VK	A	1.0	6.4	7.9	10.2	2.7	5.1	6.3	8.1	3.9	3.6	4.9	6.0
	B	2.4	6.0	4.9	7.7	5.6	4.3	1.7	4.7	7.7	4.3	3.5	5.5
	C	1.5	2.7	4.1	4.9	4.5	5.3	8.7	10.2	6.7	2.6	7.6	8.1

#### 2.1.3 正确度

参考CLSI C62^［17］^和《液相色谱-质谱临床应用建议》^［18］^，在低、高2个加标水平下（VA：300 ng/mL，600 ng/mL；25（OH）D_2_：7.00 ng/mL，14.00 ng/mL；25（OH）D_3_： 50.00 ng/mL，100.00 ng/mL；VE：4 500 ng/mL，9 000 ng/mL； VK： 0.70 ng/mL， 1.40 ng/mL），使用加标回收试验对方法的正确度进行验证，每个样本重复测定3次，计算回收率，回收率=（加标试样测定值-试样测定值）/加标量×100%。结果如表6所示，3种LC-MS/MS法检测脂溶性维生素的加标回收率分别为88.86%~109.20%（VA）、90.36%~101.79%（25（OH）D_2_）、92.83%~108.37%（25（OH）D_3_）、95.25%~106.89%（VE）、88.95%~103.30%（VK）。结果表明，方法C在不同脂溶性维生素的加标回收试验中表现相对最佳，这可能与其采用固液萃取技术作为前处理方法有关。该技术通过高比表面积微孔填料的吸附作用，显著增加了待测物与萃取剂的接触界面，从而有效提升了目标化合物的萃取效率，同时降低了生物样本基质的共提取干扰。

**表 6:** 3种LC-MS/MS方法检测脂溶性维生素的加标回收率

Compound	Method	Spiked recoveries/%	
		Low level	High level
VA	A	108.80	109.20
	B	88.90	88.90
	C	103.10	102.40
25（OH）D_2_	A	101.80	98.80
	B	91.80	90.40
	C	100.60	100.10
25（OH）D_3_	A	93.60	92.80
	B	95.80	102.00
	C	99.60	108.40
VE	A	104.10	106.00
	B	95.30	102.50
	C	106.90	103.00
VK	A	99.30	100.70
	B	89.80	103.30
	C	93.70	89.00

### 2.2 方法学比对

本研究采用Passing-Bablok回归分析、Bland-Altman一致性分析及一致性相关系数（CCC）系统评估3种LC-MS/MS方法对40份血清样本中VA、25（OH）D、VE、VK的一致性。以CCC分别计算两种试剂盒的一致性^［19］^，该系数评估观测对之间是否与穿过原点的45°线足够一致，CCC<0.900，一致性较差；0.900~0.940，一致性一般；0.950~0.990，一致性良好；CCC>0.990，一致性极好^［20］^。使用Passing-Bablok回归分析分别比较两种试剂盒之间的相关性；计算回归方程（*y*=*ax*+*b*），包括比例误差（*a*，斜率）和恒定误差（*b*，截距）的95%置信区间（CI）^［21］^；若Passing-Bablok回归中斜率的95%置信区间包含1，可认为不存在显著的比例误差；若截距的95%置信区间包含0，可认为不存在显著的系统误差。Bland-Altman作图分别比较两种试剂盒的相关性^［22］^。

目前临床上以血清25（OH）D水平（包括25（OH）D_3_和25（OH）D_2_）作为评估人体维生素D营养状态的指标^［23］^。在本研究的方法学比对中，使用总25（OH）D浓度作为参照指标，评估不同检测方法间的一致性。3种检测方法比对的相关参数如表7所示。一致性分析显示：方法A与方法C对维生素D的检测一致性为一般水平（CCC： 0.938），而对于其他脂溶性维生素的检测，各方法的一致性结果表现出较大变异（CCC： 0.322~0.853）。Passing-Bablok回归分析表明：检测VA和VE时，方法B和方法C的斜率95%CI包含1；检测VK时，方法A和方法C的斜率95%CI包含1，其他脂溶性维生素在使用3种方法检测时斜率的95%CI不包含1，表明不同方法间存在比例误差；同时，方法A和方法C检测VA、VE时截距的95%CI不包含0，检测25（OH）D时，方法B和方法C截距的95%CI也不包含0，提示不同方法在检测脂溶性维生素时存在系统误差。Bland-Altman分析结果显示：3种方法检测脂溶性维生素的平均偏差为-10.495%~40.774%（VA）、-17.746%~25.154%（25（OH）D）、-15.221%~31.845%（VE）、-46.856%~34.397%（VK）。根据正确度验证计划，4种脂溶性维生素检测偏差的可接受范围为±20%，本研究中，不同LC-MS/MS方法间存在较大的检测偏差。该偏差可能来源于方法学的差异，即不同方法所使用色谱柱规格、流动相组成及梯度洗脱程序等各不相同，这些参数可能影响目标分析物的保留行为、分离选择性及离子化效率，从而对结果的一致性造成影响。此外，3种方法所采用的校准品来源不同，且无法溯源至统一标准。这种溯源体系的差异是导致检测结果偏差的重要原因，其引起的校准偏差可能在方法间形成系统性的结果偏移，降低了结果的可比性。

**表 7:** 重新校准前3种LC-MS/MS方法检测脂溶性维生素的方法学比对和一致性分析

Compound	Method	Intercept （95%CI）	Slope （95%CI）	Correlation coefficient （95%CI）	Averagedeviation/%	Concordance consistency coefficient （95%CI）
VA	A-B	-30.577 （-64.097-5.016）	1.586 （1.505-1.667）	0.978 （0.958-0.988）	40.774	0.322 （0.219-0.418）
	A-C	-105.51 （-202.754--30.615）	1.596 （1.432-1.824）	0.922 （0.856-0.958）	30.557	0.399 （0.274-0.512）
	B-C	-34.241 （-91.425-7.020）	0.984 （0.888-1.119）	0.923 （0.859-0.959）	-10.495	0.798 （0.677-0.877）
25（OH）D	A-B	-0.135 （-2.632-2.419）	0.835 （0.764-0.897）	0.950 （0.906-0.973）	-17.746	0.824 （0.727-0.889）
	A-C	-1.973 （-3.986-0.002）	1.154 （1.089-1.221）	0.981 （0.965-0.990）	7.496	0.938 （0.899-0.962）
	B-C	-3.527 （-7.429--0.357）	1.430 （1.324-1.580）	0.950 （0.907-0.973）	25.154	0.688 （0.563-0.782）
VE	A-B	-0.354 （-1.552-0.844）	1.429 （1.303-1.560）	0.939 （0.888-0.968）	31.845	0.559 （0.432-0.665）
	A-C	-1.084 （-2.319--0.002）	1.282 （1.186-1.396）	0.947 （0.901-0.972）	16.823	0.774 （0.674-0.847）
	B-C	-0.389 （-1.613-0.441）	0.877 （0.811-1.000）	0.919 （0.852-0.957）	-15.221	0.806 （0.698-0.879）
VK	A-B	0.004 （-0.135-0.134）	0.605 （0.492-0.724）	0.847 （0.728-0.917）	-46.856	0.530 （0.382-0.651）
	A-C	-0.005 （-0.157-0.138）	0.870 （0.716-1.051）	0.807 （0.662-0.894）	-12.785	0.853 （0.753-0.915）
	B-C	0.121 （-0.218-0.313）	1.326 （1.063-1.665）	0.802 （0.654-0.891）	34.397	0.641 （0.479-0.761）

### 2.3 重新校准

相关研究表明，使用统一的校准品进行重新校准可以改善由校准偏差引起的一致性差异^［24，25］^。因此，本研究选用3种LC-MS/MS方法，对5个不同浓度的新鲜血清样本进行检测，以各方法在对应浓度下的检测均值作为目标靶值，构建新的回归校准曲线，并据此对原始检测结果进行理论校准。随后，系统比较重新校准后3种方法检测结果的相关性差异，相关参数见表8。与表7相比，校准后3种LC-MS/MS方法的相对偏差显著改善，不同方法间的平均偏差明显减小：VA的平均偏差为-1.246%~0.802%，25（OH）D的平均偏差为-0.637%~1.126%，VE的平均偏差为-0.950%~5.380%，VK的平均偏差为-8.250%~4.876%。此外，3种方法之间的一致性也有所提升，除方法B和C在检测25（OH）D与VE，以及方法A和C在检测VK时外，其他脂溶性维生素检测的回归斜率置信区间均包含1，表明校准后各方法间具有良好的一致性。同时，VA的一致性相关系数为0.946~0.983，25（OH）D为0.935~0.979，VE为0.918~0.954，VK为0.804~0.877，进一步表明方法间一致性的改善。上述结果表明，采用统一校准品可有效改善脂溶性维生素在LC-MS/MS检测中的方法间一致性。因此，建议试剂制造商重视检测结果的标准化和量值溯源，优先采用可溯源至统一参考物质的校准品，并持续优化方法学参数，以提高不同方法检测结果的一致性和可比性。

**表 8:** 重新校准后3种LC-MS/MS方法检测脂溶性维生素的方法学比对和一致性分析

Compound	Method	Intercept （95%CI）	Slope （95%CI）	Correlation coefficient （95%CI）	Average deviation/%	Concordance consistency coefficient （95%CI）
VA	A-B	-0.904 （-25.718-26.537）	0.988 （0.938-1.039）	0.978 （0.958-0.988）	-1.246	0.983 （0.968-0.991）
	A-C	43.317 （-15.677-82.534）	0.907 （0.822-1.035）	0.937 （0.881-0.967）	-0.519	0.946 （0.900-0.971）
	B-C	49.337 （-5.228-87.305）	0.911 （0.829-1.033）	0.942 （0.891-0.970）	0.802	0.946 （0.900-0.971）
25（OH）D	A-B	2.096 （-0.290-4.607）	0.932 （0.851-1.000）	0.950 （0.906-0.973）	1.126	0.944 （0.898-0.970）
	A-C	-0.820 （-2.787-1.166）	1.033 （0.975-1.092）	0.981 （0.965-0.990）	0.490	0.979 （0.961-0.988）
	B-C	-4.403 （-7.491--1.453）	1.151 （1.061-1.247）	0.950 （0.907-0.973）	-0.637	0.935 （0.885-0.964）
VE	A-B	1.325 （0.456-2.238）	0.940 （0.856-1.027）	0.939 （0.888-0.968）	5.380	0.950 （0.913-0.972）
	A-C	-0.175 （-1.133-0.700）	1.062 （0.984-1.151）	0.947 （0.901-0.972）	4.438	0.954 （0.919-0.974）
	B-C	-1.412 （-2.997--0.420）	1.099 （1.018-1.260）	0.919 （0.852-0.957）	-0.950	0.918 （0.860-0.952）
VK	A-B	-0.101 （-0.321-0.547）	1.033 （0.853-1.250）	0.846 （0.726-0.916）	-8.250	0.859 （0.753-0.922）
	A-C	-0.343 （-0.559--0.099）	1.339 （1.105-1.606）	0.797 （0.643-0.889）	-3.302	0.877 （0.793-0.929）
	B-C	-0.079 （-0.417-0.125）	1.188 （0.935-1.511）	0.786 （0.625-0.882）	4.876	0.804 （0.663-0.889）

此外，本研究结果显示，不同LC-MS/MS方法在VK检测中的一致性较低，且在重新校准后未见明显改善。这可能与当前VK质谱检测的技术局限性有关。VK在生物样本中的内源性浓度较低且具有较高的疏水性，检测过程中易受基质效应干扰^［26］^，因此对检测系统的灵敏度及前处理方法的特异性提出了更高要求。大气压化学电离（APCI）源因其在疏水性化合物电离中的技术优势，被认为是VK质谱检测的优选离子源^［27］^。然而，目前多数临床质谱检测平台多配置ESI源，且部分仪器未集成APCI模块，或需通过硬件切换实现APCI模式转换，增加了操作复杂性。本研究采用的检测方案基于ESI，检测VK时测得的离子抑制率为58.6%~94.8%，这可能是3种试剂盒在校准后一致性改善效果有限的另一原因。

## 3 结论

本研究评估了临床3种LC-MS/MS方法在脂溶性维生素检测中的分析性能和一致性。结果表明，3种LC-MS/MS方法的检测性能均满足临床要求，但在VK等脂溶性维生素的检测中仍需进一步优化方法学。此外，3种LC-MS/MS方法检测结果的一致性偏低，可能与校准偏差有关。采用统一校准品进行重新校准可显著提高LC-MS/MS方法检测结果的一致性。
